# Tumor-associated macrophages in glioblastoma: implications for immunotherapy

**DOI:** 10.3389/fimmu.2026.1949078

**Published:** 2026-10-09

**Authors:** Jianan Lei, Mei Lao, Zhuona Ni, Wenhao Chai, Manrong Fang, Zhuzhen Wen, Xuan Lv, Yuxin Gao, Kairui Hu

**Affiliations:** 1 The First People’s Hospital of Jiande, Hangzhou, Zhejiang, China; 2 School of Basic Medical Sciences, Zhejiang Chinese Medical University, Hangzhou, Zhejiang, China; 3 The Third Clinical Medical School, Zhejiang Chinese Medical University, Hangzhou, Zhejiang, China; 4 The Fourth Clinical Medical College, Zhejiang Chinese Medical University, Hangzhou, China; 5 Guangzhou Pearl-River Vocational College of Technology, Guangzhou, China; 6 The First Affiliated Hospital of Zhejiang Chinese Medical University(Zhejiang Provincial Hospital of Chinese Medicine), Hangzhou, China

**Keywords:** blood-brain barrier, CSF1R inhibitor, glioblastoma, immunotherapy, metabolic reprogramming, single-cell transcriptomics, tumor microenvironment, tumor-associated macrophage

## Abstract

Glioblastoma, IDH-wildtype, CNS WHO grade 4 (GBM) is the most aggressive primary malignant brain tumor in adults, with a median overall survival of approximately 15 months despite maximal surgical resection, radiotherapy, and chemotherapy with temozolomide. This therapeutic resistance is increasingly attributed to the immunosuppressive tumor microenvironment (TME), within which tumor-associated macrophages (TAMs) represent the most abundant infiltrating immune cell population. GBM-resident TAMs originate from two distinct lineages: embryonic brain-resident microglia and bone marrow-derived monocytes. They exhibit substantial phenotypic and functional heterogeneity beyond the classical inflammatory versus immunosuppressive dichotomy. Recent advances in single-cell RNA sequencing and spatial transcriptomics have identified multiple TAM states with distinct functional and spatial features, including GPNMB^+^ and VSIG4^+^ marker-defined subsets as well as a mesenchymal transcriptional program predominantly associated with monocyte-derived macrophages, each preferentially enriched in distinct spatial niches with substantial overlap and plasticity, and driving context-dependent immunosuppression through distinct metabolic and signaling pathways. Mechanistically, TAMs promote GBM progression via metabolic reprogramming, cytokine-mediated functional adaptation, and direct suppression of adaptive immunity. TAMs are also directly implicated in resistance to temozolomide and immunotherapies, including immune checkpoint inhibitors and chimeric antigen receptor T cell (CAR-T) therapy. This review comprehensively summarizes recent mechanistic insights and translational advances in TAM-targeted strategies, including TAM depletion via CSF1R inhibition, functional reprogramming toward inflammatory phenotypes, metabolic intervention, and nanoparticle-mediated precision drug delivery capable of penetrating the blood-brain barrier. Unlike earlier reviews that focused solely on TAM biology, this study explicitly connects the spatial distribution and functional heterogeneity of TAM subpopulations to therapeutic vulnerability, providing a practical framework for designing biomarker-driven, patient-specific combination immunotherapy trials.

## 1 Introduction

Glioblastoma, IDH-wildtype, CNS WHO grade 4 (GBM) is the most common and fatal primary malignant brain tumor in adults, accounting for approximately 45% to 48% of all primary malignant central nervous system neoplasms (1, 2). Despite decades of therapeutic innovation, the clinical prognosis for GBM patients remains poor. After maximal safe surgical resection, concurrent radiotherapy, and adjuvant temozolomide chemotherapy, median overall survival is only 14 to 16 months (3, 4). Given that GBM recurs in nearly all patients and rapidly develops therapeutic resistance, there is an urgent need to elucidate the biological mechanisms underlying treatment failure and to develop more effective therapeutic strategies (5, 6).

The tumor microenvironment (TME) plays a critical role in driving GBM progression, allowing tumor cells to evade immune surveillance and develop treatment resistance (7, 8). The GBM TME is a highly immunosuppressive milieu composed of malignant glioma cells, infiltrating immune populations, stromal cells, endothelial cells, and extracellular matrix components (9, 10). Within this complex ecosystem, tumor-associated macrophages (TAMs) are the most abundant infiltrating immune cell population, accounting for 30% to 50% of the total tumor mass and serving as key regulators of immunosuppression (11, 12).

TAMs in GBM originate from two ontogenetically distinct lineages—brain-resident microglia and bone marrow-derived monocytes—and exhibit substantial phenotypic and functional heterogeneity that transcends traditional classification systems, as recently uncovered by single-cell and spatial transcriptomic studies (13, 14). Their functional states are shaped by converging metabolic cues and cytokine axes within the TME, which collectively drive TAM-mediated immunosuppression, angiogenesis, and therapeutic resistance (15–17). These mechanistic details are elaborated in Section 2.

Importantly, TAMs are critically involved in mediating resistance to conventional and emerging immunotherapies. They contribute to the poor response to immune checkpoint inhibitors (ICIs) in GBM clinical trials and impair the efficacy of chimeric antigen receptor (CAR)-T cell therapy by creating an immunosuppressive microenvironment that restricts effector cell infiltration and persistence (18–20). The identification of pathogenic subpopulations of human glioma-associated macrophages linked to accelerated glioma progression further highlights the clinical impact of TAM heterogeneity on patient outcomes (20).

Given the central role of TAMs in GBM pathogenesis, targeting this population has emerged as a promising approach to improve therapeutic efficacy. Current TAM-targeted strategies include CSF1R/CCR2-mediated depletion, functional reprogramming toward inflammatory phenotypes, metabolic interventions, and nanoparticle-based precision delivery capable of penetrating the blood-brain barrier (21–23). Preclinical studies have demonstrated the promise of these approaches, especially when combined with immune checkpoint blockade (18, 19, 24), as summarized in **Figure 1**. However, significant barriers—including TAM heterogeneity, adaptive resistance, and the physical challenge of crossing the blood-brain barrier—still impede clinical translation (21, 25). Integrating single-cell and spatial omics into clinical decision-making may enable patient-specific TAM profiling and inform precision immunotherapy (23, 26), while next-generation cellular platforms such as CAR-NK, CAR-M, and CAR-γδ T cells offer alternative strategies that may bypass the limitations of conventional CAR-T therapy in GBM (22, 27).

**Figure 1:**
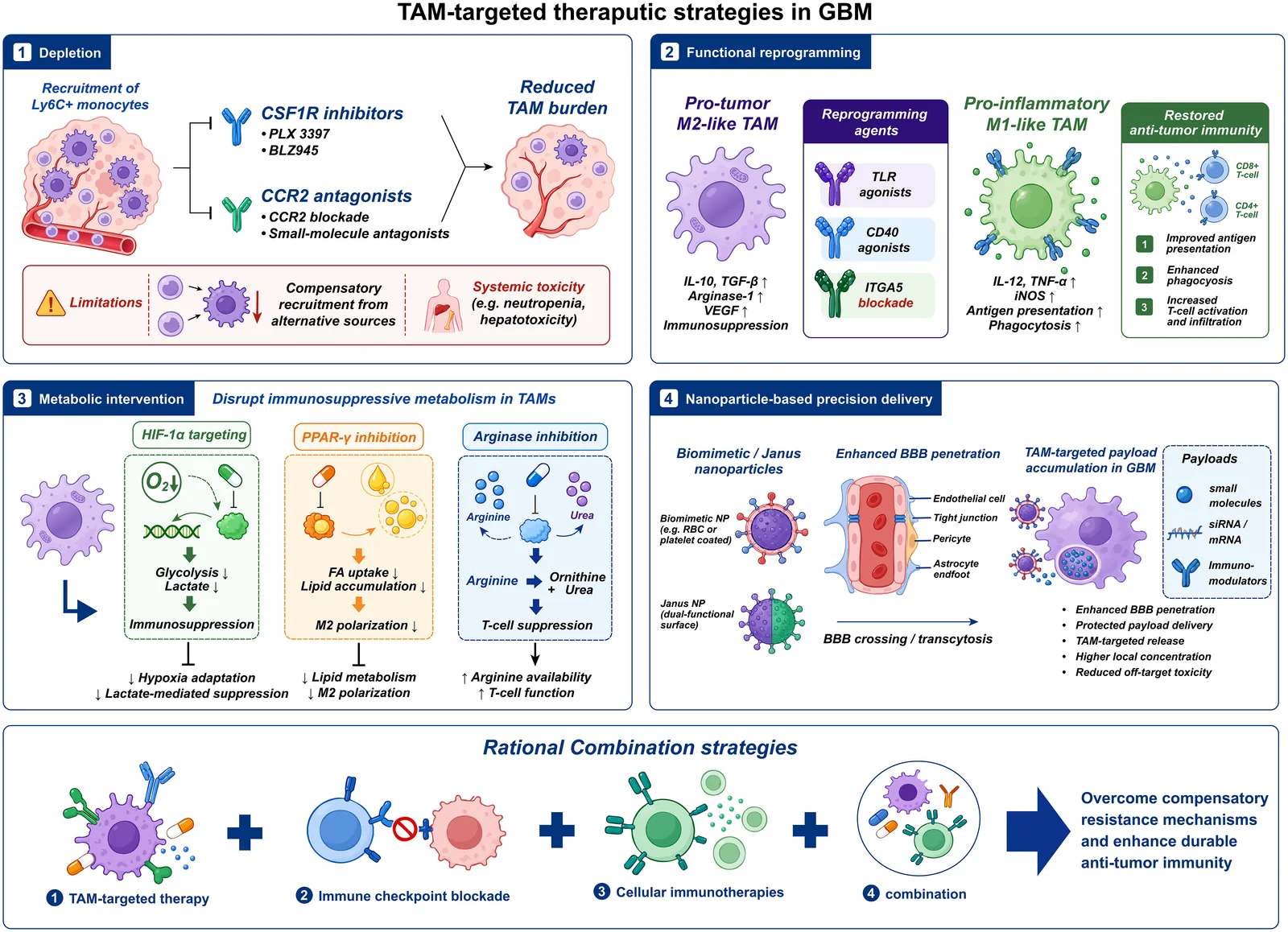
TAM-targeted therapeutic strategies in GBM. Four major intervention approaches are currently under investigation: (1) TAM depletion via CSF1R inhibitors (PLX3397, BLZ945) and CCR2 antagonists, which reduce TAM burden but are limited by compensatory recruitment and systemic toxicity; (2) functional reprogramming of immunosuppressive, tumor-supportive TAM states toward inflammatory, antigen-presenting functional programs using TLR agonists, CD40 agonists, or ITGA5 blockade, which harness macrophage plasticity to restore anti-tumor immunity; (3) metabolic interventions targeting PPAR-γ, arginase, or HIF-1α pathways to disrupt TAM immunosuppressive metabolism; and (4) nanoparticle-based precision delivery systems, including biomimetic and Janus nanoparticles, which enhance blood-brain barrier penetration and enable TAM-specific payload accumulation. Rational combinations of these approaches with immune checkpoint blockade and cellular immunotherapies are being explored to overcome compensatory resistance mechanisms.

In this review, we synthesize current mechanistic insights into the origins, heterogeneity, and functional roles of GBM-associated TAMs, with particular emphasis on their contributions to immunosuppression and therapeutic resistance. We also delineate the evolving landscape of TAM-targeted therapeutic strategies—encompassing depletion, reprogramming, metabolic intervention, and nanotechnology-mediated delivery—while critically assessing the challenges and limitations associated with each approach. This review extends prior work by establishing a heterogeneity-to-therapy matching framework that links distinct TAM states—defined by ontogeny (microglia versus monocyte-derived), spatial niche (perivascular versus hypoxic core), and surface markers (e.g., GPNMB^+^, VSIG4^+^)—to therapeutic vulnerabilities, providing a practical translational scaffold for designing biomarker-stratified combination immunotherapy trials (28).

## 2 Biology of tumor-associated macrophages in glioblastoma

### 2.1 Origins and heterogeneity

Tumor-associated macrophages in glioblastoma arise from two fundamentally distinct lineages, with differing contributions to the formation of an immunosuppressive milieu and the development of therapeutic resistance (29, 30). Brain-resident microglia originate from primitive yolk-sac progenitors during embryogenesis, and are maintained by local self-renewal in adulthood; circulating monocytes contribute very little to this population under normal physiological conditions (31). In contrast, bone marrow-derived monocytes are recruited to the GBM microenvironment through chemokine signaling pathways, particularly the CCL2/CCR2 and CXCL12/CXCR4 axes, and differentiate into macrophages following tumor extravasation (32, 33). Recent single-cell transcriptomic analyses have shown that these two populations have distinct transcriptional profiles and functional specializations within GBM: microglia-derived TAMs are preferentially enriched at the invasive margin and peritumoral regions, whereas monocyte-derived TAMs accumulate in hypoxic tumor core regions (34, 35) (**Figure 2**).

**Figure 2:**
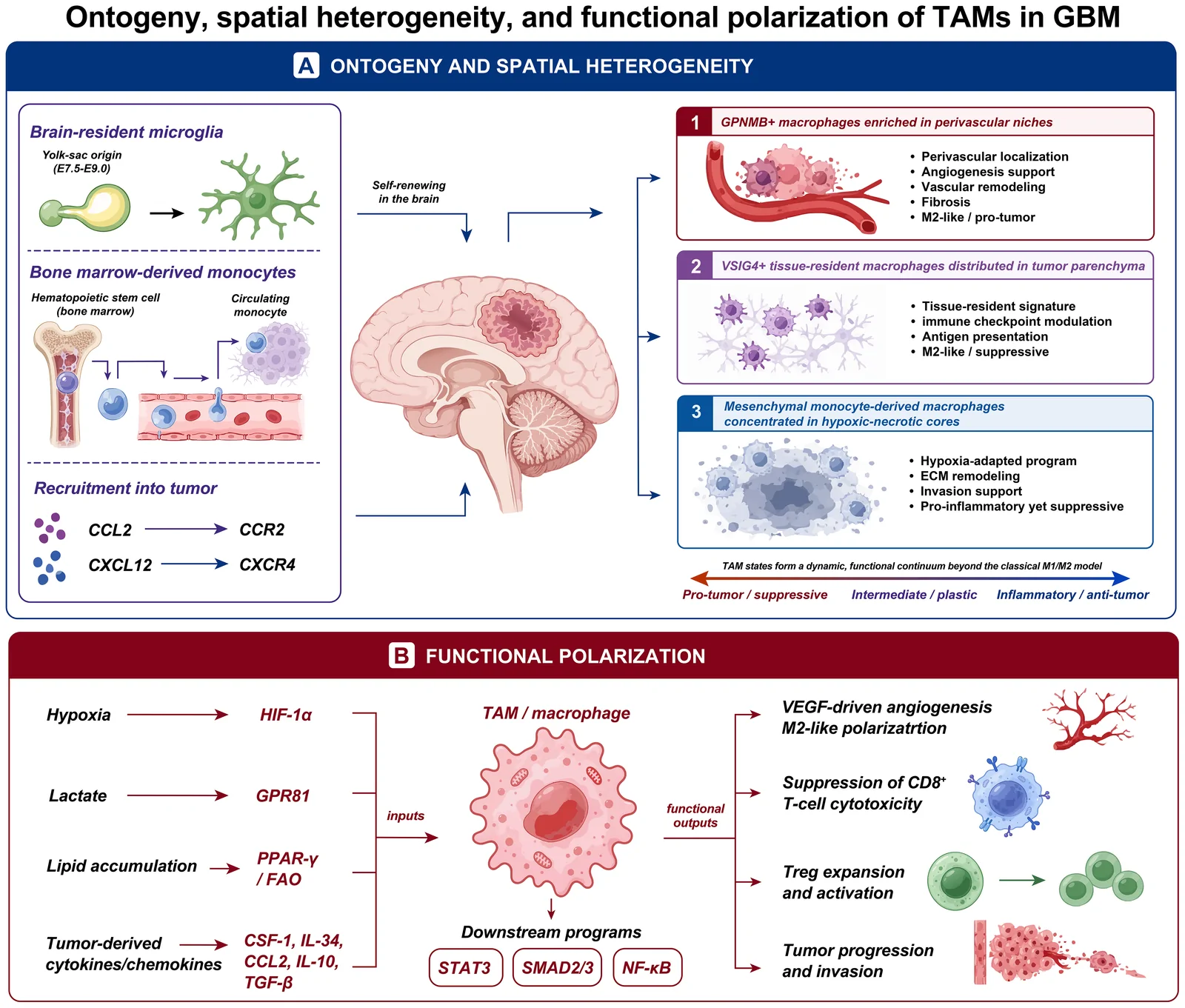
Ontogeny, spatial heterogeneity, and functional adaptation of TAMs in GBM. **(A)** TAMs originate from two distinct lineages: embryonic yolk sac-derived microglia (green) and bone marrow-derived monocytes (blue) recruited via CCL2/CCR2 and CXCL12/CXCR4 signaling. These populations give rise to overlapping, marker- and program-defined TAM states that are preferentially enriched in distinct niches: GPNMB^+^ macrophages preferentially localize to perivascular regions, VSIG4^+^ macrophages are distributed throughout the tumor parenchyma, and the mesenchymal transcriptional program is enriched in monocyte-derived macrophages of the hypoxic-necrotic core. These states are not mutually exclusive and exhibit substantial plasticity and context dependence. **(B)** TAM functional adaptation is driven by converging metabolic and cytokine-mediated signals. Hypoxia stabilizes HIF-1α, promoting VEGF-driven angiogenesis and immunosuppressive functional adaptation. Lactate activates GPR81 signaling, while lipid accumulation engages PPAR-γ and fatty acid oxidation pathways. Tumor-derived CSF-1, IL-34, CCL2, IL-10, and TGF-β coordinate downstream STAT3, SMAD2/3, and NF-κB signaling to establish immunosuppressive transcriptional programs that suppress CD8^+^ T cell cytotoxicity, promote Treg expansion, and drive tumor progression.

While early studies often categorized TAMs into broadly defined immunosuppressive or inflammatory groups, it is now evident that these binary descriptions fail to capture the remarkable phenotypic complexity of GBM-associated TAMs. Accumulating evidence supports the view that TAM phenotypes exist along a dynamic continuum influenced by local environmental signals, rather than fitting into a rigid binary framework (13, 36). Single-cell RNA sequencing has uncovered multiple TAM subpopulations with distinct functional profiles. It is important to recognize, however, that these populations are defined at different biological levels. GPNMB^+^ and VSIG4^+^ are marker-defined subsets that can originate from either microglia or monocytes depending on the tumor context, whereas mesenchymal macrophages represent a functionally-defined lineage state predominantly derived from bone marrow-derived monocytes and enriched in hypoxic niches. With this hierarchical distinction in mind, key illustrative subsets include: GPNMB^+^ TAMs, often localized to perivascular niches, have been shown to engage COL6A3^+^ fibroblasts in vascular fibrosis (37); VSIG4^+^ TAMs, distributed throughout the tumor parenchyma, suppress T cell cytotoxicity through checkpoint molecule expression (38, 39). By contrast, the mesenchymal transcriptional program—enriched in monocyte-derived macrophages within hypoxic-necrotic cores—represents a distinct functional state defined by a specific transcriptional regulatory network (TNF/CELSR2/p65/Kla-HDAC1/EPAS1 axis) rather than by a single surface marker (15). Thus, while GPNMB^+^ and VSIG4^+^ subsets can originate from either lineage, the mesenchymal state is predominantly lineage-associated. This hierarchical distinction informs the therapeutic framework discussed in Section 3. It is important to note, however, that the functional characterization of VSIG4^+^ macrophages remains a subject of ongoing debate. While the prevailing view emphasizes their immunosuppressive and pro-tumorigenic roles (39), the possibility of context-dependent functional heterogeneity—including potential antitumor activities under specific microenvironmental conditions—cannot be excluded and warrants further investigation. This functional dichotomy—further discussed in Section 6—underscores the limitations of assigning fixed functional labels to TAM subpopulations and highlights the need for spatially resolved functional studies that can distinguish between protective and pathogenic VSIG4^+^ states *in situ*. Spatial transcriptomic analyses further demonstrate that these subpopulations occupy specialized niches within the tumor: GPNMB^+^ macrophages are enriched in perivascular regions, whereas mesenchymal macrophages predominate in hypoxic-necrotic core areas, highlighting how spatial context critically shapes TAM function and therapeutic vulnerability (40, 41), as summarized in **Figure 2**. The key characteristics of these illustrative functional axes are summarized in **Table 1**.

**Table 1:** Key characteristics of clinically relevant TAM states in GBM.

Feature	GPNMB^+^ macrophages	VSIG4^+^ macrophages	Mesenchymal monocyte-derived macrophages
Defining level	Marker-defined subset	Marker-defined subset	Lineage + functional state
Defining markers	GPNMB, COL6A3 interaction	VSIG4, S100A10	TNF, CELSR2, EPAS1
Primary ontogeny	Mixed (microglia and monocyte-derived, context-dependent)	Marker-defined subset; ontogeny context-dependent (microglia- or monocyte-derived)	Bone marrow-derived monocytes
Spatial niche	Preferentially enriched in perivascular regions	Distributed throughout tumor parenchyma	Preferentially enriched in hypoxic-necrotic core
Key functional program	Vascular fibrosis; ECM remodeling	T cell suppression via checkpoint molecules	Temozolomide resistance, mesenchymal transition
Proposed therapeutic vulnerability	Anti-angiogenic agents; ECM modulators	Checkpoint-based reprogramming (anti-VSIG4, anti-PD-1)	Metabolic interventions (HIF-1α, FAO inhibitors)
Evidence level	Hypothesis; not directly tested in GBM models	Experimentally demonstrated in preclinical models (38)	Experimentally demonstrated in preclinical models (15)
**Key references**	(37)	(38, 39)	(15)

These entities are defined at different biological levels; GPNMB^+^ and VSIG4^+^ are surface marker-defined subsets that can arise from either ontogenic lineage, whereas the mesenchymal state is defined by a lineage-associated transcriptional program. They are presented together for their clinical relevance rather than as a rigid taxonomic classification. Entries labeled “Hypothesis” represent author-proposed therapeutic vulnerabilities that have not been directly tested in GBM models; entries labeled ‘Experimentally demonstrated in preclinical models’ are supported by direct experimental evidence in GBM models.

Bold values indicate the defining marker or key feature of each TAM state.

### 2.2 Recruitment and functional adaptation mechanisms

The recruitment and functional adaptation of TAMs in GBM are orchestrated by a complex interplay of cytokine signaling, chemokine gradients, and metabolic pressures within the tumor microenvironment (7, 20). Tumor-derived CSF-1 and IL-34 support macrophage recruitment, survival, and differentiation through CSF1R signaling, establishing a principal axis for TAM maintenance in GBM (21, 42). In parallel, CCL2-mediated CCR2 signaling and CXCL12/CXCR4 chemotaxis guide monocyte trafficking from the periphery into the tumor parenchyma, a process further amplified by tumor hypoxia and vascular endothelial activation (43, 44).

Metabolic reprogramming is a critical determinant of TAM functional adaptation in GBM. Tumor hypoxia stabilizes HIF-1α signaling in macrophages, enhancing VEGF expression and favoring pro-angiogenic phenotypes (16, 45). Lactate released by glycolytically active glioma cells promotes immunosuppressive TAM functional adaptation via GPR81-dependent signaling and HIF-1α activation, while simultaneously suppressing inflammatory cytokine production (7, 46). Furthermore, lipid accumulation and oxidized fatty acids reinforce immunosuppressive macrophage states through PPAR-γ and fatty acid oxidation-associated metabolic pathways (46, 47). In GBM specifically, GPNMB^+^ lipid-laden macrophages have been shown to acquire myelin debris via CD36-mediated phagocytosis and transfer lipids to mesenchymal-like glioma cells through the LXR/ABCA1 axis, thereby directly fueling tumor growth (48). Of note, cancer cell-derived arginine fuels polyamine biosynthesis in tumor-associated macrophages, establishing a metabolic crosstalk that facilitates immune evasion and mesenchymal transition (17, 49). These metabolic-immune interactions thus provide a mechanistic rationale for targeting TAM metabolism therapeutically (**Figure 2**). Importantly, these metabolic programs are not uniformly distributed across TAM subpopulations: GPNMB^+^ macrophages are preferentially sustained by lipid metabolism pathways within perivascular niches, whereas mesenchymal monocyte-derived macrophages rely on hypoxia-adapted glycolytic and glutaminolytic metabolism within necrotic cores, underscoring the subpopulation-specific metabolic vulnerabilities discussed in Section 3.

Cytokine-mediated signaling converges on transcriptional programs that dictate TAM functional states. IL-10 and TGF-β activate STAT3 and SMAD2/3 pathways, inhibiting antigen presentation and inflammatory activation; conversely, IFN-γ and Toll-like receptor ligands activate NF-κB and STAT1 signaling, which promotes inflammatory, antigen-presenting functional programs in TAMs (8, 13). Additionally, the myeloid biomarker MS4A6A drives an immunosuppressive microenvironment in glioblastoma through activation of the PGE2 signaling axis, underscoring the complexity of TAM regulatory networks (42), as illustrated in **Figure 2**.

### 2.3 Functional roles in GBM progression

TAMs drive glioblastoma progression by promoting tumor growth, invasion, angiogenesis, and therapeutic resistance (8, 11). These tumor-promoting functions are not uniformly distributed across the TAM compartment but are enriched in specific spatial niches and transcriptional states, as detailed in Section 2.1 and summarized in **Table 1** (15, 37, 38). Through the secretion of growth factors such as EGF, PDGF, and TGF-β, TAMs directly stimulate glioma cell proliferation and survival (9). Additionally, TAM-derived matrix metalloproteinases and cathepsins degrade extracellular matrix components, facilitating tumor cell invasion into the surrounding normal brain parenchyma (10, 50). TAMs also potently promote tumor angiogenesis through the production of VEGF, angiopoietin, and IL-8, processes that are particularly pronounced in the hypoxic tumor core where TAM density is highest (51, 52).

The immunosuppressive functions of TAMs constitute a major barrier to effective anti-tumor immunity in GBM. TAMs suppress CD8^+^ T cell cytotoxicity through multiple mechanisms, including PD-L1 upregulation, arginase-1-mediated L-arginine depletion, and secretion of immunosuppressive cytokines such as IL-10 and TGF-β (53, 54). Beyond the PD-1/PD-L1 axis, TAMs in GBM express or regulate additional immune checkpoint molecules that contribute to T cell exhaustion. B7-H3 (CD276) is markedly upregulated on glioma cells and tumor vasculature and is detected in the majority of primary and recurrent GBM specimens; through engagement with incompletely characterized counter-receptors, B7-H3 preferentially dampens T helper type 1-mediated immune responses, and its blockade enhances CD8^+^ T cell infiltration and therapeutic efficacy in GBM models (55–57). B7-H4 (VTCN1) is induced on glioma-associated macrophages and microglia via IL-6/IL-10-mediated STAT3 activation; B7-H4^+^ myeloid cells preferentially accumulate at the peritumoral edge, suppress T cell responses, and are associated with poor prognosis in glioma patients (58). VISTA (PD-1H), an inhibitory checkpoint preferentially enriched in glioma-associated myeloid cells, has been implicated in glioma immunosuppression. Single-nucleus and spatial transcriptomic analyses link VISTA/VSIG3-associated interactions to microglia and monocyte-derived macrophage compartments and to adverse outcomes in GBM (59). Mechanistically, PD-1H/VISTA promotes suppressive myeloid polarization, glioma cell growth and invasion, CD8+ T-cell dysfunction and shorter survival in preclinical glioma models (60), and VISTA blockade or VISTA/PD-1 dual blockade prolongs survival in orthotopic GBM models (60, 61). The original PD-1H/VISTA-deficiency experiments attributed tumor resistance in glioma models, including a radiotherapy setting, to a CD4-dependent mechanism (62). Importantly, VISTA should not be interpreted as a uniformly microglia-specific marker: recent single-cell, lineage, spatial and organoid studies indicate that glioma myeloid immunomodulatory programs are driven predominantly by microenvironmental cues rather than ontogeny alone (63). Thus, VISTA is best framed as a myeloid-enriched, context-dependent checkpoint whose expression and function vary with tumor stage, treatment pressure and spatial niche. The TIGIT/CD155 axis represents another emerging pathway: CD155 is co-expressed with PD-L1 on suppressive myeloid cells and glioma cells in human GBM tissue, and its engagement with TIGIT on infiltrating T cells impairs effector function and promotes regulatory T cell differentiation (64). Consistently, dual targeting of TIGIT and PD-1 markedly prolongs survival in preclinical GBM models compared with either blockade alone (65). Collectively, these axes expand the repertoire of TAM-mediated immunosuppression beyond PD-L1 and offer additional opportunities for combination immunotherapy. Beyond their direct effects on CD8^+^ cytotoxic T lymphocytes, TAMs engage in multifaceted crosstalk with other immunosuppressive and stromal compartments in the GBM ecosystem. First, TAMs potently promote regulatory T cell (Treg) expansion and functional maturation through the secretion of TGF-β and IL-10, which together sustain FoxP3 expression and Treg-mediated suppression of effector responses (28). This TAM-Treg axis operates in a positive feedback loop, as Treg-derived signals further reinforce immunosuppressive TAM functional adaptation, creating a mutually reinforcing immunosuppressive circuit (66). Second, TAMs cooperate closely with myeloid-derived suppressor cells (MDSCs) to orchestrate a coordinated myeloid barrier against antitumor immunity. Both populations share overlapping chemokine receptors and immunosuppressive effector mechanisms, including arginase-1-mediated L-arginine depletion, reactive oxygen species (ROS) production, and PD-L1 upregulation, collectively attenuating T cell infiltration and function in the GBM microenvironment (34). Third, emerging evidence has established a bidirectional communication loop between TAMs and glioma-instructed astrocytes. Astrocytes activated by tumor-derived signals acquire an immunosuppressive phenotype that depends, at least in part, on TAM-mediated mechanisms. TAMs respond to astrocyte-derived factors including IL-6 and CCL2, further amplifying local immunosuppression (67). Fourth, glioma-neuronal circuit remodeling, recently identified as a driver of regional immunosuppression, appears to operate via TAM-dependent pathways. Neuronal activity modulates chemokine expression within the tumor microenvironment, thereby influencing TAM recruitment and functional adaptation in a spatially restricted manner (33). Collectively, these interconnected networks extend TAM-mediated immunosuppression beyond direct T cell inhibition, establishing a complex web of cellular interactions that sustain immune evasion and therapeutic resistance in GBM. Moreover, TAMs impair dendritic cell maturation and promote regulatory T cell expansion, collectively establishing a comprehensive immunosuppressive network (15, 68).

Critically, TAMs are intimately implicated in resistance to both standard-of-care and emerging therapies. TAM-derived factors protect glioma cells from temozolomide-induced DNA damage and apoptosis, while mesenchymal monocyte-derived macrophages enhance temozolomide resistance through the TNF/CELSR2/p65/Kla-HDAC1/EPAS1 axis (15). In the context of immunotherapy, TAMs create a hostile microenvironment that impedes CAR-T cell infiltration and persistence, and spatial transcriptomic analyses have revealed that TAM-rich regions correspond to areas lacking response to PD-1 blockade in recurrent GBM (18, 19, 24). The identification of a pathogenic subpopulation of human glioma-associated macrophages linked to glioma progression underscores the clinical relevance of TAM heterogeneity in shaping therapeutic outcomes (69).

## 3 TAMs as immunotherapy targets

The therapeutic targeting of TAMs in GBM has historically proceeded along strategy-centric lines-depletion, reprogramming, metabolic modulation, or delivery optimization-without sufficient consideration of the TAM subpopulation being targeted. However, the emerging single-cell and spatial transcriptomic landscape of GBM-associated TAMs compels a paradigm shift: rather than applying a uniform therapeutic approach to all TAMs, rational treatment selection should be guided by the dominant TAM subpopulation composition within each tumor. This framework is based on the premise that therapeutically relevant vulnerabilities should be mapped to the defining features of each TAM state, rather than to a fixed set of three subpopulations. Specifically: (1) GPNMB expression identifies TAMs engaged in perivascular fibrosis and ECM remodeling, which may be susceptible to interventions disrupting angiogenic signaling or extracellular matrix turnover, rather than to conventional CSF1R inhibition (37); (2)VSIG4 expression marks TAMs with checkpoint-mediated T cell suppression, representing a logical target for immune checkpoint-based reprogramming strategies, although caution is warranted given their potential context-dependent antitumor functions (38, 39); and (3) the mesenchymal transcriptional program in monocyte-derived macrophages, associated with hypoxia and temozolomide resistance, may be best targeted by metabolic interventions that disrupt hypoxia-adapted survival pathways (15). This feature-to-therapy matching framework does not imply that all three states co-exist as rigidly defined subpopulations in every tumor, but rather provides a conceptual scaffold for interpreting clinical trial outcomes, designing biomarker-stratified trials, and ultimately guiding patient-specific therapeutic decisions as TAM profiling technologies become increasingly accessible.

### 3.1 TAM depletion strategies

Given the central role of TAMs in GBM pathobiology, therapeutic strategies aimed at depleting these cells have garnered substantial interest. CSF1R inhibition represents the most extensively investigated approach. Preclinical studies using the brain-penetrant CSF1R inhibitor BLZ945 demonstrated that CSF1R blockade reduces TAM burden, shifts macrophage functional state transition toward an antitumor phenotype, and delays tumor progression in orthotopic GBM models (70). These findings established the therapeutic rationale for targeting TAMs in glioma, although subsequent work revealed that resistance can emerge through compensatory mechanisms (48). PLX3397 (pexidartinib), a small-molecule CSF1R inhibitor, has shown efficacy in reducing TAM numbers and delaying tumor progression in orthotopic GBM models (71). However, clinical translation has been hampered by compensatory mechanisms, including increased granulocyte infiltration and elevated CSF-1 levels that sustain residual TAM populations (12, 72). These depletion strategies, along with reprogramming and approaches, are illustrated in **Figure 1**.

CCR2 antagonism offers an alternative depletion strategy by blocking monocyte recruitment from the periphery. Preclinical studies indicate that CCR2 blockade reduces monocyte-derived TAM infiltration and enhances the efficacy of standard therapies (32, 43). Nevertheless, CCR2 inhibition spares brain-resident microglia, which may compensate for the loss of monocyte-derived macrophages and sustain immunosuppression (28). The incomplete nature of TAM depletion, coupled with potential toxicities including systemic macrophage depletion and impaired wound healing, underscores the limitations of this approach and has prompted investigation of alternative strategies that preserve macrophage viability while redirecting their function (73, 74). Despite the promising preclinical rationale, the clinical translation of CSF1R inhibitors in GBM has yielded modest results. In the completed Phase II trial (NCT01349036), the CSF1R inhibitor PLX3397 (pexidartinib) demonstrated limited single-agent activity in recurrent GBM, with an objective response rate below 5% in the evaluable patient cohort (75). The most frequently observed adverse events included fatigue, elevated transaminases, and dysgeusia, with dose-limiting toxicities primarily attributed to off-target kinase inhibition and systemic macrophage depletion (75). To our knowledge, no peer-reviewed clinical trial data for CCR2 antagonists in GBM have been published to date. Preclinical evidence from murine models indicates that CCR2 blockade reduces monocyte-derived TAM infiltration (32, 43, 76), although clinical translation remains unverified. Resistance to CSF1R inhibition in clinical settings has been linked to compensatory CSF-1 upregulation, increased granulocyte infiltration, and sustained TAM survival through alternative pathways, including IL-34 and CXCL12/CXCR4 signaling (13). Importantly, resistance mechanisms are not uniform across all CSF1R inhibitors. While PLX3397-treated patients frequently exhibit compensatory myeloid remodeling, BLZ945 resistance has been associated with PI3K pathway reactivation in residual macrophage populations, suggesting that inhibitor-specific resistance profiles may inform sequential or combination therapeutic strategies (77). Collectively, these clinical observations underscore the need for rational combination regimens and biomarker-driven patient selection rather than the broad application of TAM depletion as monotherapy. Among these strategies, CSF1R inhibition depletes both lineages, but its impact is relatively more pronounced on microglia-derived TAMs due to their greater dependence on CSF1R signaling, whereas CCR2 antagonism selectively limits monocyte-derived macrophage infiltration. Notably, these depletion strategies exhibit subpopulation-selective effects: CSF1R inhibition predominantly affects microglia-derived TAMs, while CCR2 antagonism specifically targets monocyte-derived macrophages, sparing brain-resident microglia, which may continue to express markers such as GPNMB or VSIG4 and maintain immunosuppressive functions that may require alternative therapeutic approaches as outlined in the following sections.

### 3.2 TAM reprogramming and state acquisition

Functional reprogramming of TAMs toward inflammatory, anti-tumor phenotypes has emerged as a conceptually attractive alternative to depletion, offering the potential to harness macrophage plasticity for therapeutic benefit while avoiding the safety concerns associated with global macrophage elimination (78, 79). Small molecule and antibody-based approaches targeting TAM state acquisition have shown promise in preclinical GBM models. TLR agonists, IFN-γ, and CD40 agonists activate NF-κB and STAT1 signaling, promoting inflammatory, antigen-presenting functional programs in TAMs (80, 81). Blocking integrin alpha-5 (ITGA5) potentiates the efficacy of anti-PD-1 therapy on glioblastoma by remodeling tumor-associated macrophages, demonstrating the feasibility of combination approaches that integrate TAM reprogramming with immune checkpoint blockade (26).

Metabolic interventions represent a particularly innovative dimension of TAM reprogramming. Given the central role of metabolic reprogramming in TAM state acquisition, strategies targeting glycolysis, fatty acid oxidation, and amino acid metabolism offer opportunities to reshape TAM function (16, 46). Inhibition of PPAR-γ or disruption of arginine-polyamine metabolic crosstalk may shift TAMs toward inflammatory states, while modulation of hypoxia-HIF-1α signaling could reduce pro-angiogenic TAM phenotypes (17, 49). The integration of metabolic-targeted TAM reprogramming with immune checkpoint inhibitors or CAR-based cellular therapies may yield synergistic anti-tumor effects (5, 25). Within this framework, mesenchymal monocyte-derived macrophages-which are metabolically adapted to hypoxic conditions-may be particularly amenable to metabolic interventions targeting HIF-1α or fatty acid oxidation pathways, whereas VSIG4^+^ tissue-resident macrophages may be better targeted by checkpoint-based reprogramming strategies.

### 3.3 TAM-targeted drug delivery

The blood-brain barrier (BBB) and blood-tumor barrier (BTB) pose formidable challenges for systemic delivery of TAM-targeted therapeutics to GBM (27, 82). Nanotechnology-based platforms have emerged as promising solutions, enabling targeted drug delivery to TAMs while minimizing systemic toxicity. Dual-targeting zein Janus nanoparticles have been shown to reprogram tumor-associated macrophages for enhanced glioblastoma therapy, achieving preferential accumulation in TAM-rich tumor regions (22). Genetically engineered biomimetic nanoparticles enable synergistic activation of glioma-associated macrophages against glioblastoma, leveraging macrophage membrane camouflaging to enhance BBB penetration and tumor targeting (23).

Ferritin-armed extracellular vesicles with enhanced BBB penetration and tumor-targeting ability have demonstrated efficacy in preclinical GBM models, delivering therapeutic payloads specifically to TAMs within the tumor microenvironment (83). Ultrasound-assisted brain delivery of nanomedicines further enhances BBB permeability, enabling improved therapeutic access to intratumoral TAMs (84). These precision delivery strategies hold particular promise for targeting specific TAM subpopulations, thereby maximizing therapeutic efficacy while minimizing off-target effects (85). For example, nanoparticle platforms can be engineered to preferentially accumulate in perivascular niches where GPNMB^+^ macrophages reside, or to penetrate hypoxic tumor cores where mesenchymal macrophages are enriched, offering subpopulation-specific delivery opportunities that align with the heterogeneity-to-therapy framework proposed in this review.

### 3.4 Challenges and limitations

Despite substantial preclinical progress, TAM-targeted therapies face significant challenges in clinical translation (**Table 2**). The pronounced heterogeneity of TAMs, encompassing multiple subpopulations with distinct origins, spatial distributions, and functional specializations, complicates the development of broadly effective interventions (34, 40). Beyond TAM heterogeneity, the durability of TAM-targeted therapies is critically limited by multiple layers of compensatory resistance that operate at the cellular, metabolic, and tissue levels. First, monocyte depletion strategies, including CCR2 antagonism and anti-CCL2 antibodies, frequently trigger compensatory activation and expansion of brain-resident microglia, which rapidly acquire immunosuppressive functions that phenocopy those of depleted monocyte-derived macrophages, thereby preserving the overall immunosuppressive burden within the tumor (77, 86). This microglial compensation is partially mediated by enhanced CSF-1 and IL-34 signaling within the TME, sustaining microglial survival even when peripheral monocyte recruitment is blocked (77). Second, metabolic feedback compensation is a major resistance mechanism. When one metabolic pathway is therapeutically disrupted, such as arginine-polyamine biosynthesis, TAMs upregulate alternative fuel sources, including fatty acid oxidation and glutamine metabolism, to sustain their immunosuppressive functions, effectively bypassing the intended metabolic blockade (16). This metabolic plasticity is underpinned by transcription factor networks, including PPAR-γ and HIF-1α, which coordinate adaptive metabolic rewiring in response to nutrient and signaling perturbations (16). Third, tumor-secreted chemokines dynamically remodel the myeloid compartment after therapeutic intervention. CSF1R inhibition, for example, triggers a compensatory surge in CCL2, CCL7, and CXCL12 production by glioma cells and stromal fibroblasts, which in turn recruit alternative myeloid populations, including neutrophils and inflammatory monocytes, that partially restore immunosuppressive functions despite effective CSF1R blockade (76, 77). Collectively, these layered compensatory mechanisms explain the frequent failure of monotherapeutic TAM depletion and underscore the need for combination regimens that simultaneously target multiple axes of TAM maintenance and recruitment. Tumor adaptive mechanisms, including compensatory recruitment of alternative immune suppressor cells and upregulation of alternative survival pathways, may limit the durability of TAM-targeted responses (6, 87). Furthermore, the dynamic plasticity of TAMs enables rapid functional state transition in response to therapeutic pressure, potentially restoring immunosuppression following initial treatment response (50).

**Table 2:** Comparison of TAM-targeted therapeutic strategies in glioblastoma.

Strategy	Representative agents	Mechanism	Key preclinical findings	Clinical status and observations	Primary limitations
CSF1R Inhibition	PLX3397, BLZ945	CSF1R blockade reduces TAM survival	Reduced TAM burden; delayed tumor progression in orthotopic GBM models	PLX3397: Phase II (NCT01349036) in recurrent GBM: PFS6 8.6%, no objective responses. BLZ945: Phase I/II (NCT02829723) was terminated with no results posted	Compensatory CSF-1/IL-34 upregulation; granulocyte influx; systemic macrophage depletion
CCR2 Blockade	PF-04136309, MLN1202	Inhibition of monocyte recruitment	Reduced monocyte-derived TAM infiltration	No peer-reviewed GBM-specific clinical trial data. PF-04136309 evaluated in pancreatic cancer only. MLN1202 evaluated in bone metastases (NCT01015560)	Spares brain-resident microglia, which may compensate functionally; incomplete depletion of TAM pool
TAM Reprogramming	TLR agonists, CD40 agonists, anti-ITGA5	Functional redirection from immunosuppressive to inflammatory states	Enhanced CD8+ T cell infiltration and anti-tumor immunity in preclinical models	Preclinical in GBM; Phase I/II trials ongoing in other solid tumors; no GBM-specific clinical data available to date	Phenotypic instability; risk of off-target inflammatory toxicity; context-dependent efficacy
Metabolic Intervention	PPAR-γ inhibitors, arginase inhibitors, HIF-1α modulators	Disruption of TAM metabolic support pathways	Altered TAM polarization; reduced production of immunosuppressive metabolites	Preclinical; no GBM-specific clinical data available	Systemic metabolic effects; target selectivity challenges; BBB penetration
Nanoparticle	Zein Janus NPs; biomimetic NPs; ferritin-armed EVs	Targeted payload delivery across BBB	Enhanced BBB penetration; TAM-specific accumulation; improved anti-tumor efficacy in preclinical models	Preclinical; several formulations under development	Manufacturing complexity; batch-to-batch variability; scalability; long-term safety

BLZ945 trial (NCT02829723) was terminated with no results posted. PLX3397 data are from Butowski et al., 2016 (PFS6 8.6%, no objective responses).

### 3.5 Clinical prognostic value of TAM-related biomarkers

Beyond therapeutic targeting, TAM-associated markers have been increasingly evaluated for their prognostic utility in patient cohorts. A growing body of retrospective analyses has correlated the abundance of specific TAM subpopulations with overall survival (OS) and progression-free survival (PFS). For instance, high VSIG4^+^ macrophage infiltration, as assessed by multiplex immunohistochemistry in resected GBM specimens, has been associated with shorter OS in multiple independent cohorts, with multivariate analyses identifying VSIG4 density as an independent negative factor (39). Similarly, elevated expression of the myeloid biomarker MS4A6A correlates with reduced PFS and diminished response to standard chemoradiotherapy, potentially reflecting its role in PGE2-driven immunosuppression (42). Conversely, the prognostic significance of GPNMB^+^ macrophages remains less consistent, with some studies reporting an association with aggressive disease features, while others failing to demonstrate independent prognostic value after adjusting for established clinical covariates (20, 37). Beyond single-marker analyses, integrated TAM signatures comprising multiple subpopulation-specific genes have shown improved prognostic stratification in transcriptomic datasets, suggesting that composite scores may outperform individual markers in predicting clinical outcomes (34). Notably, the prognostic impact of TAM markers is frequently context-dependent, influenced by intrinsic tumor features such as IDH mutation status, MGMT promoter methylation, and spatial localization within the tumor core versus the invasive margin (88). These findings highlight the potential of TAM subpopulation profiling to complement existing molecular stratification systems and inform patient selection for TAM-directed therapies in future clinical practice.

## 4 Insights from single-cell and spatial omics

The advent of single-cell RNA sequencing and spatial transcriptomics has revolutionized our understanding of TAM heterogeneity and its implications for GBM immunotherapy (35, 89). Single-cell analyses have confirmed and extended the heterogeneity of GBM-associated TAMs, identifying cells expressing GPNMB or VSIG4, or exhibiting mesenchymal-like transcriptional signatures, across multiple datasets (37, 69). However, whether these marker-defined observations represent transcriptionally equivalent entities across different studies, platforms, and species remains to be established through harmonized reference atlases (90–92). Beyond confirming their existence, single-cell and spatial omics approaches have further refined our understanding of these populations by revealing their transcriptional regulatory networks, inter-clonal relationships, and dynamic transitions between functional states (15, 38, 41).

Spatial transcriptomics has provided critical insights into the spatial organization of TAM subpopulations within the GBM ecosystem. TAMs in the tumor core, characterized by hypoxia and necrosis, exhibit distinct functional profiles compared to those at the invasive margin or in perivascular regions (10, 88). This spatial heterogeneity has direct therapeutic implications: TAMs in the hypoxic core may be more susceptible to metabolic interventions targeting HIF-1α, whereas perivascular TAMs may be more amenable to therapies disrupting angiogenic signaling (16, 17). Integrated genomic and transcriptomic profiling of glioblastoma has further revealed ecDNA-driven heterogeneity and microenvironmental reprogramming, highlighting the complex interplay between genetic alterations and immune landscape evolution (89).

The application of single-cell and spatial omics to clinical specimens is increasingly informing patient stratification and treatment selection. Spatial transcriptomic analysis has revealed a lack of response to PD-1 blockade in recurrent glioblastoma, with TAM-rich regions showing minimal T cell infiltration and activation (18). Conversely, tumors with diverse TAM subpopulations and active T cell-TAM interactions may be more responsive to combination regimens integrating TAM reprogramming with immune checkpoint blockade (19, 26). These findings underscore the potential for TAM subpopulation profiling to guide precision immunotherapy in GBM. Despite these advances, several methodological challenges limit the interpretation and cross-study comparability of current single-cell and spatial omics findings. First, differences in tissue processing, dissociation protocols, and sequencing depth across studies introduce substantial technical variability that may obscure biologically meaningful TAM subpopulation signatures or generate study-specific artifacts (90, 91). Second, most published datasets are derived from surgical specimens obtained at a single time point, and the relatively small sample sizes of most spatial transcriptomic studies limit statistical power for detecting rare TAM subpopulations (93, 94). Third, the integration of single-cell and spatial data remains methodologically challenging, with current computational approaches varying widely in their alignment algorithms and cell-type deconvolution methods (95, 96).

### 4.1 Challenges in cross-study synthesis and harmonization

Beyond these technical limitations, the direct comparability of TAM subpopulation signatures across different studies remains a substantial biological and analytical challenge. First, the use of diverse preclinical models—including orthotopic xenografts, genetically engineered mouse models with distinct driver mutations (e.g., PDGFB-driven, NF1-silenced, or EGFRvIII-amplified), and patient-derived organoids—introduces substantial biological variability that complicates cross-study synthesis, as each model may generate a distinct TAM transcriptional landscape (92). Second, even when the same marker (e.g., GPNMB or VSIG4) is used to define a subpopulation, the underlying transcriptional state may differ significantly across studies due to variations in sequencing platforms, batch effects, and analytical pipelines (90, 91). Third, TAM states identified in different studies using the same marker name should not be assumed to represent identical transcriptional entities; without a unified reference atlas and standardized pipelines, a “GPNMB+ macrophage” in one dataset may correspond to a substantially different state in another, particularly in cross-species comparisons between mouse models and human GBM (92). These challenges underscore the need for multi-institutional consortia with harmonized protocols—such as those established by the Human Tumor Atlas Network—to develop consensus TAM taxonomies, standardized analytical pipelines, and validated spatial interaction networks, and to prospectively collect longitudinal specimens that can inform clinical decision-making (97).

## 5 Future perspectives

Building upon the biological and therapeutic frameworks established in Sections 2–4, several promising directions merit particular attention for advancing TAM-targeted approaches toward clinical translation. First, combination strategies integrating TAM-targeted interventions with established and emerging immunotherapies represent a logical next step. The combination of CSF1R inhibition with immune checkpoint inhibitors, CAR-T cell therapy, or radiotherapy may overcome compensatory mechanisms and achieve more durable responses (20, 21). Tumor-targeted cytokines that rescue CAR-T cell activity and engage host T cells against glioblastoma demonstrate the feasibility of such combination approaches (19).

Second, individualized treatment strategies based on TAM subpopulation composition and metabolic state hold promise for precision immuno-oncology. If future harmonized atlases validate VSIG4^+^ density or mesenchymal-like transcriptional signatures as reproducible biomarkers, tumors enriched for these features might be prioritized for checkpoint-based reprogramming or metabolic interventions, respectively (38, 50). The integration of artificial intelligence and machine learning approaches for predicting TAM function and therapeutic response from multi-omics data may further refine patient stratification. In solid tumors, multi-omics-assisted deep learning models have already identified macrophage-oriented gene modules as predictive biomarkers for immunotherapy sensitivity (98), and pathway-informed graph neural networks have been applied to decode intercellular immune signaling networks that govern treatment resistance (99). While current AI applications in neuro-oncology have primarily focused on radiographic tumor detection and classification (100, 101), the extension of these integrative computational frameworks to GBM-specific single-cell and spatial datasets—incorporating TAM subpopulation signatures, metabolic states, and spatial interaction networks—represents a critical next step toward clinically actionable predictive models.

Third, novel cellular therapy platforms offer alternatives to conventional CAR-T therapy that may be better suited to the GBM microenvironment. CAR-NK cells, CAR-M macrophages, and CAR-γδ T cells each offer distinct advantages in terms of tumor penetration, cytokine profile, and resistance to TAM-mediated suppression (24, 25). Harnessing ferroptosis to transform glioblastoma therapy and surmount treatment resistance represents an additional innovative approach that may circumvent TAM-mediated immunosuppression (102).

Notably, TAM-mediated resistance to CAR-T therapy operates at two mechanistically distinct layers: prior to tumor entry, perivascular TAMs remodel the stromal and vascular microenvironment to constrain CAR-T cell extravasation and infiltration; once CAR-T cells reach the tumor parenchyma, intratumoral TAMs impair their persistence and cytotoxic function through PD-L1/PD-1 engagement, arginase-1-mediated L-arginine depletion, and secretion of IL-10 and TGF-β. These TAM-dependent barriers, acting in parallel with tumor-intrinsic resistance mechanisms such as antigen heterogeneity and target loss, collectively explain the limited efficacy of CAR-T therapy in GBM clinical studies and underscore the rationale for combining CAR-T cells with TAM-directed agents.

Finally, the development of more sophisticated *in vitro* and *in vivo* model systems is essential for advancing TAM-targeted therapies. *In vitro* blood-brain barrier models, microphysiological systems, and genetically engineered mouse models that accurately recapitulate human GBM TAM heterogeneity will facilitate preclinical evaluation of novel therapeutic strategies (82, 103). Liquid biopsies for monitoring TAM dynamics and treatment response may enable real-time adaptation of therapeutic regimens (104).

## 6 Controversies and unresolved issues

Despite substantial progress, several fundamental controversies persist in TAM biology in GBM. The most prominent unresolved question concerns the net functional contribution of VSIG4^+^ macrophages. As outlined in Section 2.1, the possibility of context-dependent functional heterogeneity cannot be excluded (38, 39). Resolving this paradox will require spatially resolved functional assays and lineage-tracing models that can distinguish between opposing VSIG4^+^ states *in situ*. The discrepancies may partly reflect differences in tumor stage at which samples were analyzed (105), antibody clones used for detection, or spatial regions sampled (106). Second, the optimal therapeutic strategy for TAM targeting remains debated; while depletion offers a straightforward approach, it carries risks of systemic toxicity and compensatory rebound, whereas reprogramming strategies aim to exploit macrophage plasticity but face challenges related to phenotypic stability and off-target inflammation (107). Third, the relative contributions of microglia and monocyte-derived macrophages to GBM progression and therapeutic resistance remain incompletely defined, with conflicting reports across different preclinical models and patient cohorts (108). Fourth, the generalizability of findings from immunocompetent mouse models to human GBM is frequently called into question, given the marked differences in immune repertoire, tumor evolution kinetics, and TAM transcriptional states between species (109). Critically, resolving these controversies will require not only better preclinical models but also systematic efforts to reconcile discrepant findings. As discussed in Section 4, reconciling these discrepancies will require coordinated multi-institutional efforts to benchmark TAM subpopulation definitions across platforms, harmonize spatial and single-cell data pipelines, and systematically compare TAM responses to therapeutic interventions across model systems (92, 97). Moving forward, such harmonized efforts will be essential to translate TAM biology into clinically actionable knowledge (97).

## 7 Conclusion

Tumor-associated macrophages (TAMs) are central orchestrators of immunosuppression, tumor progression, and therapeutic resistance in glioblastoma (GBM). Their marked heterogeneity, encompassing distinct ontogenetic origins, spatial niches, metabolic programs, and functional specializations, poses both challenges and opportunities for therapeutic intervention. The integration of single-cell and spatial omics technologies has transformed our understanding of this heterogeneity, enabling the rational design of biomarker- driven and patient-specific therapeutic strategies. While TAM depletion strategies have yielded limited clinical efficacy due to compensatory mechanisms, emerging approaches centered on functional reprogramming, metabolic modulation, and precision drug delivery offer renewed promise. Moving forward, the success of TAM-targeted immunotherapy will depend on mechanism-informed combination regimens that integrate TAM-directed interventions with immune checkpoint blockade, cellular therapies, and metabolic modulators, guided by comprehensive TAM state and spatial profiling of the tumor microenvironment. This framework, which explicitly links TAM heterogeneity to therapeutic vulnerability, provides a practical roadmap for designing next- generation precision immunotherapy trials for GBM.
